# Editorial: thematic issue on interactions across microbial ecosystems

**DOI:** 10.1093/femsec/fiaf031

**Published:** 2025-04-01

**Authors:** Søren J Sørensen, Kornelia Smalla, Mette Burmølle, Itzhak Mizrahi

**Affiliations:** University of Copenhagen, Department of Biology, Copenhagen, 2100 K, Denmark; Julius Kühn Institute, Institute for Epidemiology and Pathogen Diagnostics, Messeweg 11-12, 38104 Braunschweig, Germany; University of Copenhagen, Department of Biology, Copenhagen, 2100 K, Denmark; Ben-Gurion University of the Negev, Faculty of Natural Science, Beer-Sheva, 8410501, Israel

Microorganisms are the foundation of all ecosystems, driving biogeochemical cycles, maintaining ecosystem stability, and shaping host physiology. Their interactions—with each other, with hosts, and with their environment—are central to microbial ecology. The 16th Symposium on Bacterial Genetics and Ecology (BAGECO), held in Copenhagen, Denmark, from 26 to 30 June 2023, brought together researchers from across the world to explore these complex microbial interactions. The theme of *Interactions Across Microbial Ecosystems* reflected the growing recognition that microbial communities are rarely isolated but instead dynamically interact across ecosystems. Whether in natural environments, host-associated microbiomes, or engineered systems, microbes engage in competition, cooperation, and gene exchange, with consequences for biodiversity and function. This thematic issue of *FEMS Microbial Ecology* presents seven selected studies that provide novel insights into these interactions, highlighting the breadth and interdisciplinary nature of modern microbial ecology.

Aligning with the topics of the BAGECO23 symposium, the studies featured in this issue address fundamental questions about microbial interactions in both natural and engineered ecosystems. Microbial communities in terrestrial and aquatic habitats are constantly influenced by environmental changes, including climate-driven disturbances and anthropogenic pressures. Masch et al. (2025) investigate how bark beetle infestations in *Picea abies* (Norway spruce) forests alter fungal communities across different habitats (soil, litter, and wood). By examining shifts in mycobiome diversity and function, their work highlights how tree dieback cascades through the ecosystem, with implications for nutrient cycling and forest health. This research aligns with broader efforts to understand how environmental disturbances reshape microbial communities, particularly in the context of climate change (Stursová et al. 2014, Cavicchioli et al. 2019). Similarly, Vass et al. (2024) explore microbial community coalescence in estuarine environments, demonstrating that the mixing of freshwater and marine communities enhances microbial diversity and ecological stability. Their findings contribute to the growing body of research on microbial dispersal and resilience, emphasizing that repeated mixing events may play a role in stabilizing ecosystems under environmental fluctuations (Rilling et al Jansson and Hofmockel 2020, Herren and McMahon 2017).

In host-associated microbiomes, microbial interactions influence health, development, and ecological function, but disruptions to these relationships can have far-reaching consequences (McFall-Ngai et al. 2013). Giraud et al. (2024) examine the active prokaryotic communities associated with shrimp larvae (*Penaeus stylirostris*) in aquaculture settings, revealing how environmental factors shape microbial assemblages and identifying potential probiotic strains that could contribute to improved disease resistance. Their findings align with the broader understanding of microbial dysbiosis in aquaculture, where imbalances in microbial communities can lead to increased disease susceptibility (Vadstein et al. 2018). In contrast, Bodkhe et al. (2024) investigate microbiome inheritance in *Caenorhabditis elegans*, demonstrating that dauer larvae—a stress-resistant stage—are largely devoid of gut bacteria. Their study challenges the assumption that host-associated microbiomes are continuously transmitted across generations, suggesting that stress-induced developmental transitions can act as bottlenecks in microbial inheritance and potentially influence host–microbe coevolution (Shapira 2017).

Beyond natural and host-associated ecosystems, microbial interactions are also key to engineered environments, where they mediate pollutant degradation, biofilm formation, and secondary metabolite production (Timmis et al. 2017). Martin et al. (2024) investigate microbial communities in moving bed biofilm reactors (MBBRs) under increasing micropollutant concentrations. Their findings demonstrate that while biofilm-associated prokaryotes adapt and maintain functional activity under chemical stress, numbers of protozoa decline, highlighting the differential resilience of bacteria and their protozoan grazers in wastewater treatment systems. Understanding these microbial responses is crucial for optimizing bioremediation and wastewater management strategies (Flemming et al. 2016). Meanwhile, Buijs et al. (2024) introduce *SecMet-FISH*, a fluorescence-based technique for detecting and visualizing secondary metabolite-producing microorganisms. By combining fluorescence *in situ* hybridization (FISH) with gene-targeted probes, their method provides a powerful tool for linking microbial function to phylogeny in complex environmental samples, opening new possibilities for studying microbial competition and chemical signalling in microbial communities (Foster and Bell 2012, Steen et al. 2019).

In addition to empirical studies, theoretical and conceptual perspectives remain essential for advancing microbial ecology. Geesink et al. (2024) provide an important perspective on microbial interactions, arguing that beyond direct pairwise exchanges, microbial interactions create emergent effects that shape entire ecosystems. Their work emphasizes that metabolic cross-feeding, biofilm formation, and chemical signalling generate cascading influences on microbial networks, which should be considered when studying microbial community assembly and function. These emerging secondary effects of microbial interactions have been recognized in microbiome research (Coyte et al. 2015) but require further integration into ecological and evolutionary models.

The research presented in this thematic issue reflects the diversity and dynamism of microbial ecology, spanning from host-associated microbiomes to environmental and engineered ecosystems. Collectively, these seven studies highlight the complexity of microbial interactions and emphasize the importance of interdisciplinary approaches in understanding microbial life. We extend our gratitude to the authors for their contributions and to the reviewers for their valuable feedback. We hope that this thematic issue inspires further research on the intricate web of microbial interactions that shape our world.

## References

[bib1] Bodkhe R, Trang K, Hammond S et al. Emergence of dauer larvae in *Caenorhabditis elegans* disrupts continuity of host–microbiome interactions. FEMS Microbiol Ecol. 2024;100: fiae149. 10.1093/femsec/fiae149. 39516048 PMC11590253

[bib2] Buijs Y, Geers AU, Nita I et al. SecMet-FISH: labeling, visualization, and enumeration of secondary metabolite producing microorganisms. FEMS Microbiol Ecol. 2024;100:fiae038 10.1093/femsec/fiae038. 38490742 PMC11004939

[bib3] Cavicchioli R, Ripple WJ, Timmis KN et al. Scientists' warning to humanity: microorganisms and climate change. Nat Rev Micro. 2019;17:569–86. 10.1038/s41579-019-0222-5. PMC7136171 31213707

[bib4] Coyte KZ, Schluter J, Foster KR. The ecology of the microbiome: networks, competition, and stability. Science. 2015;350:663–6. 10.1126/science.aad2602. 26542567

[bib5] Flemming HC, Wingender J, Szewzyk U et al. Biofilms: an emergent form of bacterial life. Nat Rev Micro. 2016;14:563–75. 10.1038/nrmicro.2016.94. 27510863

[bib6] Foster KR, Bell T. Competition, not cooperation, dominates interactions in microbial communities. Curr Biol. 2012;22:1845–50. 10.1016/j.cub.2012.08.005. 22959348

[bib7] Geesink P, Ter Horst J, Ettema TJG. More than the sum of its parts: uncovering emerging effects of microbial interactions in complex communities. FEMS Microbiol Ecol. 2024;100:fiae029. 10.1093/femsec/fiae029. 38444203 PMC10950044

[bib8] Giraud C, Wabete N, Lemeu C et al. Environmental factors and potential probiotic lineages shape the active prokaryotic communities associated with healthy *Penaeus stylirostris* larvae and their rearing water. FEMS Microbiol Ecol. 2024;100:fiae156. 10.1093/femsec/fiae156. 39562288 PMC11636268

[bib9] Herren CM, McMahon KD. Cohesion: a method for quantifying the connectivity of microbial communities. ISME J. 2017;11:2319–33. 10.1038/ismej.2017.94. 28731477 PMC5649174

[bib10] Jansson JK, Hofmockel KS. Soil microbiomes and climate change. Nat Rev Micro. 2020;18:35–46. 10.1038/s41579-019-0265-7. 31586158

[bib11] Martin JD, Tisler S, Scheel M et al. Total RNA analysis of the active microbiome on moving bed biofilm reactor carriers under incrementally increasing micropollutant concentrations. FEMS Microbiol Ecol. 2024;100:fiae098, 10.1093/femsec/fiae098. 38986504 PMC11385203

[bib12] Masch D, Buscot F, Rohe W et al. Bark beetle infestation alters mycobiomes in wood, litter, and soil associated with Norway spruce. FEMS Microbiol Ecol. 2025;101:fiaf015. 10.1093/femsec/fiaf015. 39890600 PMC11840958

[bib13] McFall-Ngai M, Hadfield MG, Bosch TCG et al. Animals in a bacterial world, a new imperative for the life sciences. Proc Natl Acad Sci U S A. 2013;110:3229–36. 10.1073/pnas.1218525110. 23391737 PMC3587249

[bib15] Shapira M . Host-associated microbial inheritance: mechanisms and implications for health and evolution. Nat Rev Genet. 2017;18:394–. 10.1038/nrg.2017.41.

[bib16] Steen AD, Crits-Christoph A, Carini P et al. High proportions of bacteria and archaea across most biomes remain uncultured. ISME J. 2019;13:3126–30. 10.1038/s41396-019-0484-y. 31388130 PMC6863901

[bib17] Stursová M, Baldrian P, López-Mondéjar R. Fungal community shifts and accompanying carbon cycle processes in decomposing leaves of two tree species. Microb Ecol. 2014;67:202–15. 10.1007/s00248-013-0334-7.

[bib18] Timmis KN, de Lorenzo V, Verstraete W et al. The contribution of microbial biotechnology to sustainable development goals. Microb Biotechnol. 2017;10:984–7. 10.1111/1751-7915.12818. 28840974 PMC5609250

[bib19] Vadstein O, Bergh Ø, Gatesoupe F-J et al. Microbiology and microbial ecology of fish larval gut: characteristics and implications. Aquaculture. 2019;499:149–59. 10.1016/j.aquaculture.2018.09.030.

[bib20] Vass M, Székely AJ, Carlsson-Graner U et al. Microeukaryote community coalescence strengthens community stability and elevates diversity. FEMS Microbiol Ecol. 2024;100:fiae100. 10.1093/femsec/fiae100. 39003240 PMC11287207

